# PD45 A Rapid Evidence Synthesis Method For Cancer Screening Recommendations In A Hospital Setting

**DOI:** 10.1017/S0266462324003076

**Published:** 2025-01-07

**Authors:** Carlos Muñoz-Montecinos, Catalina González-Browne, Felipe Maza, Camila Quirland

## Abstract

**Introduction:**

International agencies advocate for population-based cancer screening to prevent cancer-related deaths. The Arturo Lopez Perez Oncology Institute is interested in implementing screening programs, but international recommendations differ on program details such as screening tests, target population, age range, and frequency. A review of international evidence-based recommendations is essential for advising stakeholders on the effective implementation of screening programs.

**Methods:**

A rapid scoping review was performed to identify international recommendations on cancer screening programs. Evidence-based recommendations derived from the World Health Organization and the European Union were analyzed. We also searched for evidence-based recommendations from the following health technology assessment agencies with specific sections for evaluating screening strategies: the Canadian Agency for Drugs and Technologies in Health (Canada), the Institute for Quality and Efficiency in Health Care (Germany), the Medical Services Advisory Committee (Australia), and the National Institute of Health and Care Excellence (UK). Additionally, we explored international cancer screening programs implemented by health systems in the aforementioned countries or in countries with implemented screening programs. Finally, we searched for recommendations from scientific societies on cancer screening strategies. This iterative process was repeated for five different cancers.

**Results:**

We found a total of 32 favorable or unfavorable recommendations for breast, cervical, colorectal, lung, gastric, and prostate cancer screening. Breast and cervical cancer had the highest number of favorable recommendations, with complete agreement on the type of test and only small differences regarding age range and periodicity. On the other hand, we found some recommendations against population-based screening for prostate and gastric cancer and limited agreement for both test type and target population. Direct comparisons between the recommendations served as a guide to elaborate a cancer screening program based on the most recommended strategies.

**Conclusions:**

This rapid scoping review allowed us to assess the consistency of cancer screening recommendations. Major differences were found mainly between recommendations from international agencies and scientific societies. As a result, a cancer screening program was designed based on the most recommended strategies.

